# Post kala-azar dermal leishmaniasis in the Indian sub-continent: challenges and strategies for elimination

**DOI:** 10.3389/fimmu.2023.1236952

**Published:** 2023-08-11

**Authors:** Awnish Kumar, Vishal Kumar Singh, Rahul Tiwari, Prasoon Madhukar, Rajneesh, Shashi Kumar, Vibhav Gautam, Christian Engwerda, Shyam Sundar, Rajiv Kumar

**Affiliations:** ^1^ Centre of Experimental Medicine and Surgery, Institute of Medical Sciences, Banaras Hindu University, Varanasi, India; ^2^ Department of Medicine, Institute of Medical Sciences, Banaras Hindu University, Varanasi, India; ^3^ Immunology and Infection Laboratory, QIMR Berghofer Medical Research Institute, Herston, QLD, Australia

**Keywords:** visceral leishmaniasis, post kala-azar dermal leishmaniasis, immune regulation, IL-10, intervention strategies, elimination

## Abstract

Visceral leishmaniasis (VL) is a severe and often fatal form of leishmaniasis caused by *Leishmania donovani* in the Indian sub-continent. Post Kala-azar Dermal Leishmaniasis (PKDL) is a late cutaneous manifestation of VL, typically occurring after apparent cure of VL, but sometimes even without a prior history of VL in India. PKDL serves as a significant yet neglected reservoir of infection and plays a crucial role in the transmission of the disease, posing a serious threat to the VL elimination program in the Indian sub-continent. Therefore, the eradication of PKDL should be a priority within the current VL elimination program aimed at achieving a goal of less than 1 case per 10,000 in the population at the district or sub-district levels of VL endemic areas. To accomplish this, a comprehensive understanding of the pathogenesis of PKDL is essential, as well as developing strategies for disease management. This review provides an overview of the current status of diagnosis and treatment options for PKDL, highlighting our current knowledge of the immune responses underlying disease development and progression. Additionally, the review discusses the impact of PKDL on elimination programs and propose strategies to overcome this challenge and achieve the goal of elimination. By addressing the diagnostic and therapeutic gaps, optimizing surveillance and control measures, and implementing effective intervention strategies, it is possible to mitigate the burden of PKDL and facilitate the successful elimination of VL in the Indian sub-continent.

## Introduction

Leishmaniasis is a vector borne neglected tropical disease (NTD) caused by protozoan parasites of genus *Leishmania* and transmitted by female *Phlebotomine* sand flies (1, 2). Leishmaniasis can have different clinical manifestations including visceral leishmaniasis (VL), cutaneous leishmaniasis (CL), mucocutaneous leishmaniasis (MCL), diffuse cutaneous leishmaniasis (DCL), and post kala-azar dermal leishmaniasis (PKDL). These clinical outcomes are influenced by several factors including parasite species involved, host genetic factors and immune responses, nutritional status, as well as co-infections VL is the most severe form characterized by prolonged fever, hepatosplenomegaly (enlarged spleen and liver), weight loss, pancytopenia (decreased blood cell count) and hypergammaglobulinemia (increased antibody production), affecting major organs such as the bone marrow, liver, and spleen, and can be fatal if left untreated (3, 4). Due to the relative lack of attention and resources dedicated to leishmaniasis, the World Health Organization (WHO) classified VL as a neglected tropical disease (NTD) in 2015 (5). VL is a disease associated with poverty that manifests cyclical patterns of incidence in rural and peri-urbanization settings and can affect people of all ages.

In the Indian subcontinent, the number of reported VL cases has been consistently decreasing since 2011, nearing the elimination goal (6, 7). However, the emergence of PKDL as a sequel to VL has raised concerns. PKDL commonly manifests after apparent recovery from VL (8). While PKDL is primarily associated with *L. donovani* infection in India and Sudan, cases caused by other *Leishmania* species such as *L. infantum* or *L. tropica* have been reported in Mediterranean countries and Latin America (9). PKDL is characterized by a cutaneous rash on exposed body parts, such as the face, ears, and hands, and can also affect other areas of the body during progression (10). In some patients, PKDL appears without any prior episode of VL while inadequate treatment also increases the chances of PKDL development (11). While PKDL has relatively no mortality compared to VL, it carries significant socioeconomic implications and PKDL patients serve as a reservoir for parasites, thus contributing to parasite transmission and potential new VL cases (1, 12, 13). Active surveillance and case detection are crucial in endemic areas to achieve the goal of disease elimination. To strengthen current efforts, improved diagnostic, and treatment approaches, as well as a better understanding of immune responses in PKDL patients are required. This review aims to provide insights into the epidemiology, etiology, immunobiology, and recent advancements in the diagnosis and treatment of PKDL. Additionally, we will discuss the role of PKDL in disease transmission and its impact on the VL elimination campaign.

## Prevalence and incidence of PKDL

Most PKDL patients are concentrated in six countries (14) with India alone contributing 75% of the total cases. The remaining cases are from Sudan (9%), South Sudan (8%), Bangladesh (7%), Ethiopia (1%), and a few cases from Nepal. In India, PKDL patients mainly come from 54 districts, with 33 in Bihar, 11 in West Bengal, 4 in Jharkhand, and 6 in Uttar Pradesh (15). Although PKDL can develop before or even simultaneously with VL, it is more commonly observed after apparent cure of VL in India. In Africa, PKDL can manifest as early as 0 to 6 months after VL treatment or concurrently with VL, while in India, it typically appears as PKDL 2-3 years after treatment for VL (16). The appearance of PKDL before VL is mainly believed to be arisen from asymptomatic infections, although this has not yet been conclusively shown (17).

Despite the awareness of PKDL cases for many years, systematic data gathering only began in 2011. Since then, surveillance efforts have been intensified, leading to an increased number of recorded cases. A peak of 1982 cases was reported in 2017, and a decline in prevalence has been observed since, with only 617 PKDL cases reported in 2020 (18). PKDL has long been recognized in both Asia and Africa, particularly in regions where *L. donovani* is the primary cause of disease, and it has been hypothesized to play a potential role in the spreading of VL, especially during inter-epidemic periods (19). This hypothesis is supported by xenodiagnostic studies involving PKDL subjects, where sandflies become infected after feeding on them at a higher rate than when feeding on VL patients or individuals with asymptomatic infection, highlighting their potential importance for parasite transmission (12, 20). Therefore, active screening and treatment is crucial to prevent parasite transmission, especially in India, where only anthroponotic transmission is reported.

## Diagnosis of PKDL

The diagnosis of PKDL poses challenges due to its clinical variability and low parasite presence in certain forms of the disease. The characteristic hypopigmented macular form of PKDL is commonly misdiagnosed as vitiligo due to scanty numbers of parasites, while the nodular form of PKDL is often confused with dermatological conditions like granuloma, leprosy, secondary syphilis (13, 21–23). Both diseases can present with skin lesions, and in some cases, the skin manifestations of leprosy can resemble those of PKDL, especially the macular form. This similarity in clinical presentation can pose a challenge in distinguishing between the two conditions based solely on clinical grounds. The gold standard for diagnosis is the detection of amastigote parasites, known as Leishman-Donovan (LD) bodies, in tissue biopsies or slit-smears by microscopy. However, the sensitivity of microscopy is limited, ranging from 67-100% accuracy in nodular lesions, 36-69% in papular lesions, and 7-33% in macular lesions (24, 25).

Polymerase chain reaction (PCR)-based detection techniques, using blood, lymph node aspirates, or skin homogenates, can improve sensitivity for detecting low parasite loads, particularly in macular cases. The accuracy of PCR for detecting PKDL can range from 76 to 100% (8, 25), depending on the specific study and the target sequence used in the PCR assay. The target sequences for PCR can include different genetic and transcriptional material in the parasite genome, such as kinetoplast DNA (K DNA), ribosomal RNA, mini-exon-derived RNA (med RNA), *β-TUBULIN, and GP63* (26, 27). Nested PCR has shown higher sensitivity than conventional PCR for PKDL diagnosis (25, 27, 28).

Real-time quantitative PCR (qPCR) is a method that allows the quantification of parasite load by continuously monitoring the PCR product buildup during amplification (27, 29). It has a accuracy ranging from 91 to 100% for diagnosing PKDL (25), particularly when targeting kinetoplast DNA, when accuracy can reach 100%. qPCR can also detect very low parasite numbers, with as few as 0.0125 parasites per ml of blood being detected (30). As qPCR measures the amount of parasite DNA that correlates with parasite load, this method can provide valuable information on disease dynamics, transmission, and the potential role of asymptomatic individuals in parasite transmission. However, qPCR requires well-equipped laboratories and technical expertise, limiting its use in the field (31).

The loop-mediated isothermal amplification (LAMP) assay has gained prominence as a diagnostic method and has shown advantages over conventional PCR for the diagnosis of PKDL and other diseases, especially in field settings (13, 31–33). This assay exhibits higher sensitivity compared to conventional PCR due to its tolerance to various inhibitors present in different clinical samples (34). The LAMP assay utilizes six sets of primers that target eight regions of DNA and can amplify the DNA within a shorter time frame, typically ranging from 15 to 60 minutes, using Bst DNA polymerase (35). In various studies, the LAMP assay has demonstrated a accuracy of around 96% using kinetoplast minicircle DNA, even when only 33% of the samples were microscopically positive (31). Furthermore, a recently developed SYBR green I closed tube LAMP assay, used for the diagnosis of VL and PKDL, achieved excellent accuracy of approximately 97% with 100% specificity. This particular LAMP assay method allows for a clear visual differentiation between positive and negative results based on the color change observed (green for positive and orange for negative) without the need for additional equipment (24, 25). However, it is important to note that the utility of the LAMP assay is limited due to the possibility of false-positive results caused by cross-contamination. Additionally, there have been relatively few studies using the LAMP assay for the diagnosis of *Leishmania* infections (31).

The rk39 rapid diagnostic test (RDT) is primarily used for the detection of VL and has also been employed for the diagnosis of suspected cases of PKDL with the limitation of not being able to differentiate between past and present infections. The presence of antibodies against rk39 in PKDL cases has been observed, but the test may have limitations in terms of sensitivity and specificity when applied to PKDL diagnosis (36–38) (**Table 1**).

**Table 1:** Different diagnostic tools for PKDL, their advantages and disadvantages.

Diagnostic Methods	Advantages	Disadvantages	References
Microscopy	Gold standard method for diagnosis in short time, High specificity	Low sensitivity	(24, 25)
rK39	Rapid, non-invasive, Enhance case detection	Low sensitivity and specificity, cannot distinguish between current and past infection	(36)
PCR	Higher sensitivity (as compared to microscopy), even it can detect low parasite in sample	Time consuming process, requires trained personal and specialised laboratory setting	(8, 25)
Nested PCR	Higher sensitivity (as compared to PCR)	Second PCR depend on first PCR products	(25, 28)
Real time PCR	100% sensitivity, easily detect low number of parasites in samples, continuously monitor the PCR products with time	Require well equipped laboratories and technical expertise	(27, 30)
LAMP	Higher sensitivity as compared to Conventional PCR, amplify the DNA in short time	May give false positive result due to contamination	(31, 34)

## Treatment options for PKDL

Patients with PKDL can serve as a reservoir for *Leishmania* parasite, potentially contributing to the transmission of the disease to sandflies and posing a challenge to the control and elimination efforts of VL (12, 20, 39). To address this, suitable preventive strategies, and therapeutic options for PKDL are necessary.

PKDL lesions in most cases in Sudan tend to heal spontaneously. However, in the Indian subcontinent, PKDL can manifest in a percentage ranging from 2 to 20% of individuals who previously had VL, and it can occur months to several years after VL treatment (16). Traditionally, sodium stibogluconate (SSG) has been the treatment of choice for PKDL with standard regimen involving a daily injection of SSG at a dose of 20 milligram per kilogram (mg/kg) body weight of the patient for 20 days per month for 6 months. However, this treatment approach has several limitations, including potential toxicity, prolonged hospitalization periods, and the requirement for daily painful injections have led to challenges in its widespread use and patient compliance (27). Furthermore, due to the rise in antimony resistance in India, the use of SSG is no longer recommended for treating VL and PKDL (27, 40).

A liposomal formulation of Amphotericin B (AmBisome) has shown promising results in the treatment of PKDL. AmBisome used in six infusions of a 5 mg/kg dose over three weeks, showed a 96% cure rate in macular cases of PKDL in Sudan (41). In another study, SSG-resistant PKDL cases treated with AmBisome at a dose of 2.5 mg/kg per day for 20 days showed an 83% cure rate and resulted in regression of papular and macular lesions without any adverse events (42). A prospective cohort study in Bangladesh evaluated a short-course regimen of AmBisome at a dose of 15 mg/kg, given over 15 days in 5 biweekly infusions of 3 mg/kg. This treatment approach was efficacious, improving lesions in 89% of PKDL cases with no serious adverse events reported (43). However, concerns were raised regarding the potential emergence of hypokalemia-induced rhabdomyolysis associated with its usage (44). The effectiveness of AmBisome in treating different types of lesions in Indian PKDL patients has been studied, and it has been observed that the response to treatment can vary depending on the lesion type (45). The study found that patients with polymorphic lesions showed a more pronounced decrease in parasite burden following treatment with AmBisome compared to patients with macular lesions. These findings highlight the importance of considering the specific lesion type when choosing a treatment approach for PKDL.

Currently, two main drugs are used to treat PKDL: miltefosine and amphotericin B (AmB). Miltefosine, originally developed as an anti-cancer agent, has been repurposed for the treatment of leishmaniasis and has emerged as an important alternative for the treatment of PKDL in India (46). In India, a 12-week course of oral miltefosine with dosage adjustments based on the patient’s age and weight is the first-line treatment for PKDL (47, 48). AmB has been recommended as a second alternative for the treatment of PKDL in patients not responding to miltefosine or patients where the drug was discontinued due to toxicity. The recommended use of drug is 1mg/kg body weight with up to 60-80 doses over 4 months (49) (**Table 2**).

**Table 2:** Treatment options for PKDL.

Drug	Dosages	Side effects	References
Sodium stibogluconate (SSG)	20 mg/kg of body weight for 20 days of a month for 6 months	Cardiac toxicities, arthralgia, and prolonged hospitalization	(27, 50)
Amphotericin B	1 mg/kg for 20 to 80 days which may vary depending upon cure	Nephrotoxicity	(51, 52)
Ambisome	1mg/kg body weight with up to 60-80 doses over 4 months (India), 2.5 mg/kg per day for 20 days (Sudan), 15mg/kg over 15 days in 5 biweekly infusions of 3mg/kg (Bangladesh)	No major reported side effects	(42) (43, 49)
Miltefosine	12 weeks course and doses alter according to age and weight	Primary side effects like vomiting and diarrhoea	(48) (47)

The combination therapy of AmBisome and miltefosine has been investigated as a treatment option for PKDL and has shown promise in terms of efficacy and safety with high tolerability (53). However, it’s important to note that the use of combination therapy should be based on clinical guidelines and individual patient considerations. The decision to prescribe combination therapy should be made by healthcare professionals experienced in managing PKDL, considering factors such as the severity of the disease, the patient’s medical history, and potential drug interactions or contraindications.

## Factors that determine conversion of VL to PKDL

The factors influencing the conversion of VL into PKDL are complex and multifaceted (54). One significant factor associated with PKDL development is the use of sodium antimony gluconate (SAG) for the treatment of VL. Epidemiological and clinical data from various regions, including Sudan, Bangladesh, Nepal, and India have consistently shown an increased risk for PKDL development following SAG treatment. In Sudan, Bangladesh, and Nepal, all PKDL cases were found to have undergone SAG treatment for VL (27, 55–57). In the Indian subcontinent, where SAG resistance has emerged, approximately 73% of PKDL patients were reported to have developed the condition after SAG treatment while the remaining 27% of PKDL cases in this region were attributed to other drugs such as AmB, miltefosine or paromomycin (27, 58). In Sudan, SAG is still used for the treatment of PKDL, while in India, where there has been an increase in antimony resistance, miltefosine, AmB and AmBisome have become the primary treatment options for VL (40). Studies have shown that the use of alternative treatments such as paromomycin, miltefosine or the combination of miltefosine and AmB in VL patients reduces the likelihood of developing PKDL (59).

Immune responses and genetic factors also play a role in PKDL susceptibility. Peroxisome dysfunction due to SAG treatment has been reported to promote the development of PKDL (60). Studies have indicated that the persistence of regulatory cytokines like transforming growth factor beta (TGF-β) and interleukin 10 (IL-10) during the course of VL treatment can promote parasite persistence and contribute to PKDL development (54, 61). High levels of C-reactive protein (CRP) in the peripheral blood of Sudanese VL patients has been shown to be associated with subsequent development of PKDL (62). Similarly, another study conducted in Sudan indicated that the expression of IL-10 by keratinocytes during VL and increased circulating levels of IL-10 may be predictive for the subsequent development of PKDL (63). The elevated levels of IL-10 suggest a dysregulated immune response during VL, which may contribute to the progression to PKDL, while the expression of IL-10 by keratinocytes, which are the predominant cells in the skin, further suggests the involvement of local immune mechanisms in the development of PKDL. The precise mechanisms by which IL-10 might influence the transition from VL to PKDL requires further investigation. However, this finding highlights the important role that immunological factors may play in the development of PKDL following VL.

While genetic studies has been extensively performed on other forms of leishmaniasis, there is limited research specifically focused on PKDL (64–66). One study conducted in Sudan investigated the genetic analysis of PKDL patients and found that polymorphisms in the interferon gamma (IFN-γ) receptor gene were associated with PKDL development (67). These polymorphisms resulted in diminished responsiveness to IFN-γ, despite its higher availability. Increased IFN-γ mRNA levels, but reduced IFN-γR1 expression at both mRNA and protein levels in lesions of Indian PKDL patients has also been reported (68). This finding suggests that there may be a dysregulation in the IFN-γ signaling pathway in PKDL, leading to impaired immune responses that allow the survival of *Leishmania* parasites.

UV light exposure has been implicated as a potential risk factor for PKDL development. PKDL lesions primarily appear on sun-exposed areas of the body, suggesting a role of UV-B-induced immunosuppression and its impact on antigen presentation and immune activation (54, 69). UV-B-induced impairment of antigen presenting cells, such as epidermal langerhans cells (E-LC) (70), and modulation of cytokines like TNF-α and IL-10 in keratinocytes and lymphocytes (71, 72) may contribute to PKDL susceptibility, particularly in individual’s sensitive to UV-B. About 40% of the adult population is sensitive to UV-B which can also lead to PKDL susceptibility in cured VL subjects (54, 69). Photosensitization causes changes in the morphology and characteristic pattern of dendrites on E-LCs leading to suppressed expression of major histocompatibility complex (MHC) class II and co-stimulatory molecules affecting antigen presentation and immune activation (73, 74).

The persistence of pathogens even after clinical cure is a common characteristic of several infectious diseases (27, 75). This phenomenon can lead to the recurrence of the disease in endemic areas, as observed for leishmaniasis (76, 77). Genomic and proteomic analysis can help establish a link between disease recurrence and the persistence of the parasites by comparing current and previous parasite genotypes in the same individual. By studying the genetic and proteomic characteristics of recurrent parasites, researchers can determine whether the recurrence is due to the persistence of the same parasites or reinfection with new strains. While such studies have been conducted in murine models (54, 76), performing similar investigations in humans is logistically challenging due to the low parasite density required for establishing parasite isolates (75). However, studies in endemic areas, such as Bihar, India, have shown genetic heterogeneity in the strains isolated from VL and PKDL patients, indicating reinfection rather than persistence (77, 78). In PKDL cases, it has been observed that the disease development often triggers the reactivation of the growth of parasites from a previous VL episode. Whole genome sequencing and annotation of *Leishmania* strains in Indian PKDL patients have suggested the possibility of endosymbiotic infection or superinfection contributing to PKDL manifestations (79). Similarly, in Sudan, PKDL may develop due to the persistence of parasites, as many VL patients develop PKDL either concurrently with VL or after VL treatment (54, 80). These findings emphasize the complexity of leishmaniasis and the need for further research to understand the mechanisms underlying disease persistence, recurrence, and the development of PKDL. By elucidating the role of parasite persistence and reinfection, one can gain insights into potential strategies for improved treatment and control of leishmaniasis.

Recovery from leishmaniasis is often associated with the development of immune memory, which provides lifelong protection against reinfection in humans (81). This immune memory is crucial in conferring long-term immunity to the disease. However, certain conditions, such as immunosuppression, can impair this protective immunity, leading to the recurrence of infection (82). In VL, the *leishmania* parasites found in the liver and spleen of patients can modulate the host immune response (83). PKDL presents a fascinating challenge because despite the presence of systemic protective immunity, as evidenced by cytokine or T cell responses evaluated in whole blood assays or antigen-specific peripheral blood mononuclear cell (PBMC) responses, there is a loss of immune responses in the skin, making an individual susceptible to PKDL (84, 85). Interestingly, PKDL patients from Sudan have been found to have self-heal, likely due to the presence of higher levels of effector memory T cells in the skin. In contrast, PKDL patients from India do not self-heal (80, 81, 86, 87).

## Immunobiology of PKDL

The immunological profiles of VL and PKDL differ significantly. In VL, the cell-mediated immune (CMI) response is suppressed but is restored following treatment, leading to resistance to reinfection in most cases (88, 89). However, in PKDL, which develops in a subset of cured VL patients, there is a repressed immune response against the parasites, especially in the skin, allowing latent parasites to multiply and accumulate in the skin (16, 90).

The development of PKDL also varies between populations. In Sudan, PKDL can develop independently of VL, and the immune responses observed in PKDL patients resemble those of treated VL patients. PBMCs proliferate and secrete IFN-γ and IL-10 upon stimulation with parasite antigen (63, 85). In contrast, in the Indian subcontinent, PKDL generally develops after VL, and patients exhibit high numbers of CD8^+^ T cells in lesions and circulation, along with enhanced IL-10 production and impaired proliferation upon antigen stimulation (84, 91). These CD8^+^ T cells are also anergic, possibly due to lower expression of the co-stimulatory molecule CD28 on their cell surface (84, 92). Furthermore, reduced expression of the CD28 binding ligand CD86 on CD14^+^ monocytes suggest the presence of a skin localized immunosuppressive network in PKDL patients (84, 93).

**Figure 1:**
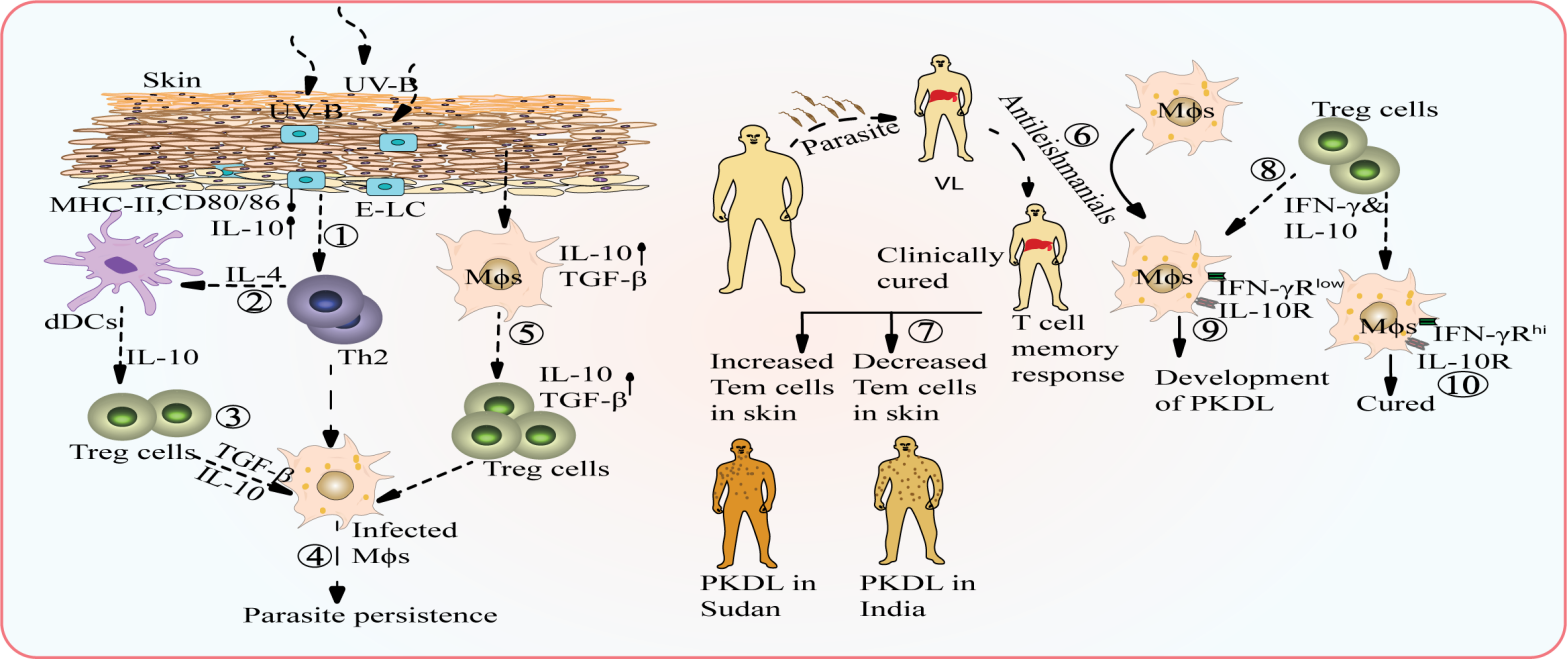
Potential interplay between environmental factors and host factors that can contribute to the conversion of VL into PKDL. UV-B radiation causes alterations in the morphology of epidermal Langerhans cells (E-LC cells) leading to suppressed expression of MHC-II and co-stimulatory molecules, but enhances the level of IL-10 (1). IL-4 secreted by Th2 cells activates dermal dendritic cells (dDCs) (2) which contribute to the cytokine pool by producing more IL-10, which creates an immunosuppressive environment and promote the expansion of regulatory T (Treg) cells (3), which further aids in parasite persistence (4). Activated macrophages secrete TGF-β, which can activate Treg cells and promote increased secretion of TGF-β, contributing to parasite persistence (5). Inadequate treatment of VL with anti-leishmanial drugs can result in the development of PKDL (6). Failure of organ-specific T cell memory response: Self-healing patients in Sudan show an increased level of effector memory T cells, indicating an enhanced immune response in the skin while PKDL patients in the Indian subcontinent have a lower titer of effector memory T cells, leading to a weak immune response and non-healing nature of the disease (7). T- cells secrete both IFN-γ and IL-10 (8) and in individuals with polymorphism in IFN-γ receptor, failure of appropriate IFN-γ signaling leads to immune suppression and dominance of IL-10 (9) while Individuals with high IFN-γR expression are least prone to developing PKDL (10).

PKDL subjects have elevated levels of IFN-γ and TNF-α, similar to VL subjects, but also exhibit simultaneous enhanced levels of immune-dampening cytokines such as IL-10 and transforming growth factor-beta (TGF-β) (68). Interestingly, despite the presence of high IFN-γ and TNF-α, Indian PKDL patients show reduced expression of receptors for these cytokines (68, 94). Similarly, Sudanese PKDL patients have been reported to possess genetic polymorphisms in the promoter region of the IFN-γ receptor 1 gene, which correlates with susceptibility to PKDL (67, 95) (**Figure 1**). Regulatory T (Treg) cells, a subpopulation of CD4^+^ T cells involved in immune homeostasis and capable of producing IL-10 during inflammation (96), are also implicated in PKDL. Elevated mRNA levels of FoxP3, CD25, and cytotoxic T lymphocyte-associated antigen 4 (CTLA-4) in skin lesions of PKDL patients suggests the accumulation of skin Treg cells in the Indian subcontinent (97). The mRNA levels of Foxp3, CD25, and IL-10 also show a direct positive correlation with the parasitic load in PKDL (97). In addition, increased plasma levels of IL-17 and IL-23, along with elevated IL-17, IL-23, and RORγt mRNA accumulation in PKDL lesions, have also been reported (98).

CD4^+^ T cells can promote pro-inflammatory responses through the production of TNF-α, IFN-γ, and IL-17, but they are also tightly regulated by Treg cells to prevent excessive inflammation and tissue damage (99). The mechanisms by which Treg cells maintain immune homeostasis are not fully understood, but certain molecules and cytokines are believed to be involved. Treg cells can produce IL-10, which has the potential to suppress excessive inflammation and tissue damage caused by pro-inflammatory responses (84, 100). Additionally, molecules like CTLA-4 and TGF-β expressed by Treg cells contribute to their suppressive function (101).

In some disease models, the secretion of IL-10 by a subset of CD4^+^ T cells called type 1 regulatory T (Tr1) cells, which also produce IFN-γ, has been observed. These Tr1 cells can suppress excessive inflammation and tissue damage. However, in certain infectious disease models, including mice infected with *Toxoplasma gondii* or *L. major*, these IL-10-producing Tr1 cells can promote the establishment and maintenance of severe infection (100, 102). Furthermore, studies on patients chronically infected with *L. donovani* have indicated that Tr1 cells are the critical source of IL-10-mediated immune suppression (103). This suggests that the balance between pro-inflammatory responses and immune regulation mediated by different subsets of CD4^+^ T cells, including Treg and Tr1 cells, is crucial for the outcome of *Leishmania* infections. However, the role of Tr1 cells in immunopathology of PKDL is still not well understood.

## Role of PKDL in transmission and potential impact on VL elimination program

PKDL patients act as a parasite reservoir for sustained disease transmission of VL within the community. Studies have shown that sand flies, the vector for *Leishmania* parasites, can become infected with the parasites by feeding on the lesions of nodular PKDL patients. This highlights the potential for PKDL patients to contribute to disease transmission (104). In the past, chronic PKDL patients have been associated with VL outbreaks, further emphasizing their role in sustaining the disease (104, 105). One challenge in addressing VL transmission is that many PKDL patients do not seek treatment as they may not exhibit symptoms other than the skin lesions, and they may appear healthy. Consequently, they can serve as a silent reservoir for the ongoing transmission of the disease within the community (80, 104).

Research has shown that sand fly infections in endemic areas are mainly contributed by active VL and PKDL cases, to a lesser extent by cured patients, and rarely by asymptomatic individuals (12, 106). Therefore, in the context of the VL elimination program, it becomes crucial to identify and track the potential parasite reservoirs, particularly PKDL patients, in order to implement enhanced vector control programs. The implementation of different sustainable vector control strategies, such as indoor residual spraying (IRS) of insecticides, insecticide-treated nets (ITNs), environmental modifications, and improved human behavior (e.g., sleeping indoors), can contribute to reducing transmission below the desired public health threshold. Currently in India alphacypermethrin (5%) is being used for IRS in houses and cattle sheds. In general, two rounds of spraying are conducted in the months of March and April as well as August and September, focusing on the villages that had reported VL cases in the last three years (107). Although it is important to focus on IRS and ITNs as insecticide-based interventions play a significant role in reducing the transmission, the challenge of insecticide resistance must be taken into consideration. Additionally, the outdoor populations of exophilic sandflies are still a concern, and can jeopardize elimination efforts.

**Figure 2:**
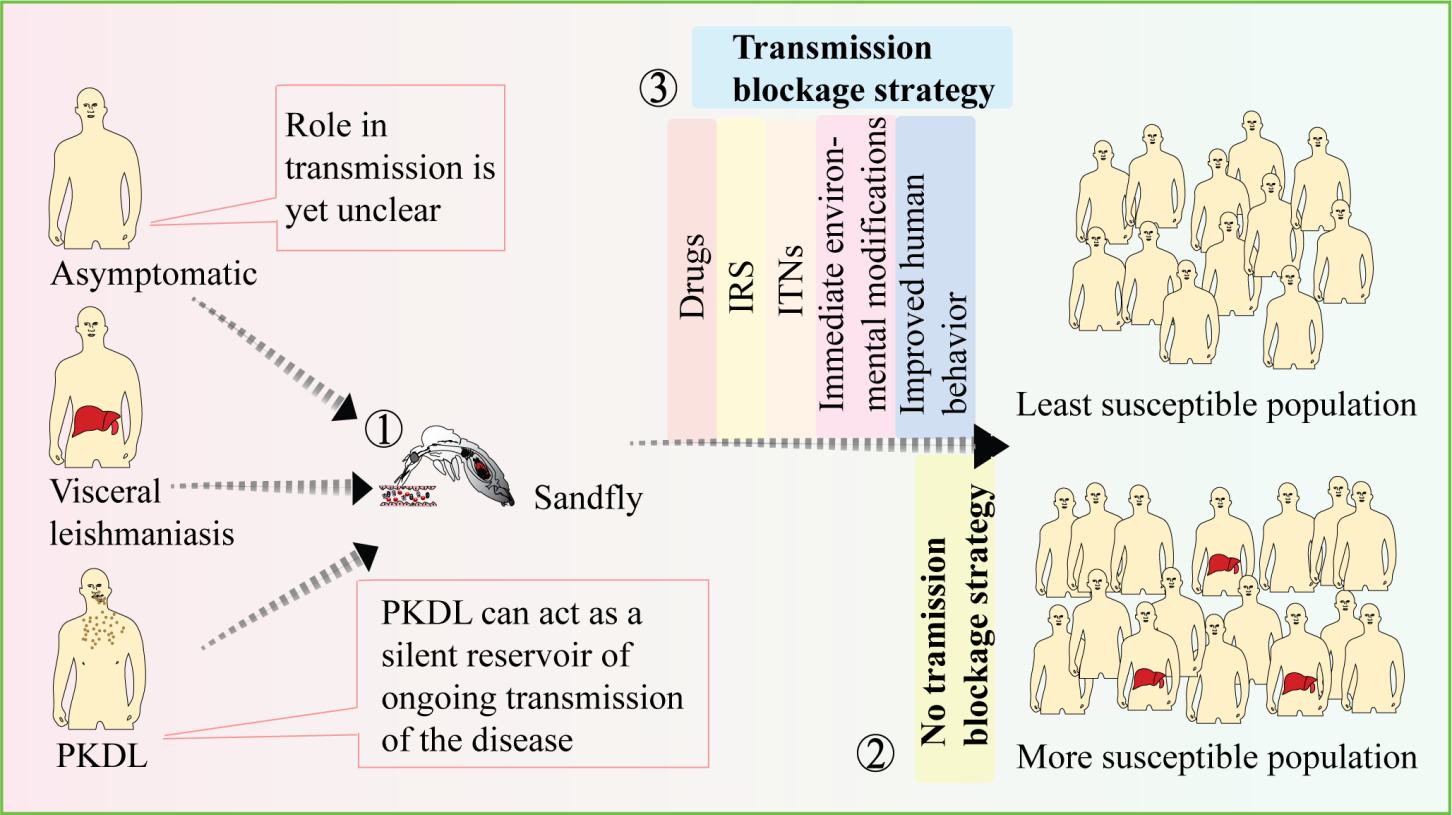
Strategies to prevent disease transmission. While the role of VL in disease transmission is well established, the contributions of PKDL and asymptomatic cases in transmission are still not fully understood (1). The presence of asymptomatic and PKDL cases can increase the vulnerability of the population to the disease (2). Therefore, employing transmission-blocking strategies such as drugs, IRS, and ITNs can be effective in preventing disease transmission and reducing population susceptibility (3). [VL-Visceral leishmania, PKDL-Post kala-azar dermal leishmaniasis, IRS-Indoor residual spraying, ITNs-Insecticide-treated nets].

These efforts combined with tracking and addressing the parasite reservoirs, are essential for achieving the goal of VL elimination (**Figure 2**). It is worth noting that in India, significant progress has been made in reducing the number of VL cases through the implementation of VL elimination programs. The number of reported VL cases has substantially decreased, with an annual incidence below the elimination threshold in a majority of blocks in the Bihar region (108). This underscores the importance of continued efforts to control transmission and address potential reservoirs to sustain the progress made towards VL elimination.

## Discussion and future prospective

PKDL is considered an immunological manifestation of VL and mainly affects populations in regions where *L. donovani* infection is highly prevalent, such as east Africa and the Indian subcontinent. It is estimated that 50-60% of PKDL cases in east Africa and 10-20% of cases in the Indian subcontinent occur in individuals who have previously been cured of VL (16). Understanding the factors that contribute to the development of PKDL is crucial. It has been proposed that inadequate treatment, environmental exposure to UV-B radiation and host genetic factors may play a role in the conversion of VL into PKDL or the appearance of PKDL alongside VL. Further research is needed to unravel the causes of PKDL and the reasons behind its occurrence in some individuals but not others. This knowledge can inform the development of targeted interventions to prevent or treat PKDL effectively. The immunological aspects of PKDL and its relationship with VL need further investigation. Understanding the immune responses during different stages of VL, PKDL, and asymptomatic periods is essential due to the involvement in host immune responses in the development of PKDL.

Additionally, the role of PKDL patients as a reservoir for leishmaniasis transmission should be considered. Even a single case of PKDL has the potential to trigger a new outbreak of VL. Therefore, continuous monitoring, early detection, and effective treatment of PKDL are crucial during the maintenance phase of VL eradication efforts. Public-health programs related to VL and PKDL prevention, recognition and treatment should be implemented through various communication initiatives in endemic areas. These initiatives can increase knowledge about the diseases and help reduce social stigma associated with them. Future research should also focus on conducting in-depth studies to understand all possible transmission scenarios and human behaviors that contribute to VL transmission. This knowledge will be instrumental in developing and implementing sustainable control strategies in endemic areas, ultimately contributing to VL elimination.

## Author contributions

AK and RK conceptualized and researched the work and wrote the paper. VS, RT and R prepared the figure. All authors reviewed and edited the manuscript.
